# Genome-scale CRISPR screen identifies host factors associated with bovine parainfluenza virus 3 infection

**DOI:** 10.1080/21505594.2025.2589554

**Published:** 2025-12-02

**Authors:** Yuanchen Geng, Chuanwen Jiang, Huaiyi Zhang, Hao Yang, Yongchong Peng, Yingyu Chen, Changmin Hu, Hailong Liu, Sheng Li, Huanchun Chen, Shengsong Xie, Aizhen Guo

**Affiliations:** a The National Key Laboratory of Agricultural Microbiology, Huazhong Agricultural University, Wuhan, China; b College of Veterinary Medicine, Huazhong Agricultural University, Wuhan, China; c Hubei Hongshan Laboratory, Huazhong Agricultural University, Wuhan, China; d Hubei International Scientific and Technological Cooperation Base of Veterinary Epidemiology, International Research Center for Animal Disease, Ministry of Science and Technology of the People’s Republic of China, Wuhan, China; e Key Laboratory of Agricultural Animal Genetics, Breeding and Reproduction of Ministry of Education & Key Lab of Swine Genetics and Breeding of Ministry of Agriculture and Rural Affairs, College of Animal Science and Technology, Huazhong Agricultural University, Wuhan, China

**Keywords:** BPIV-3, CRISPR screen, WNT5A, SLC16A13, SELENON, host factors

## Abstract

Bovine parainfluenza virus type 3 (BPIV-3) is a major pathogen associated with the bovine respiratory disease complex. However, the limited understanding of host factors crucial for BPIV-3 replication has hindered the development of effective preventive and therapeutic strategies. To tackle this critical issue, we constructed a bovine genome-wide CRISPR/Cas9 knockout library in Madin-Darby bovine kidney cells, which was then used to systematically identify and characterize the host genes essential for BPIV-3a replication. Subsequently, 10 genes were validated using both RT-qPCR and viral titration assays. Furthermore, through gene knockout or knockdown and rescue experiments, we identified three key genes required for BPIV-3a replication: Wnt family member 5A (WNT5A), solute carrier family 16 member 13 (SLC16A13), and selenoprotein N (SELENON). However, their effects on viral adhesion and internalization varied. WNT5A was involved in both processes, SLC16A13 participated solely in internalization, while SELENON had no significant impact on either. Beyond BPIV-3a, these three genes were also found to be essential for the infection of BPIV-3c and Bovine enterovirus. In conclusion, this study offers novel insights into the molecular mechanisms governing the replication and pathogenesis of BPIV-3a, BPIV-3c, and bovine enterovirus within host cells, thereby providing a foundation for identifying potential targets in the development of novel antiviral strategies.

## Introduction

Bovine parainfluenza virus type 3 (BPIV-3) is a single-stranded, negative-sense RNA virus with a genome of approximately 15 kb, belonging to the genus *Respirovirus* within the family Paramyxoviridae [1]. First isolated in the United States in 1959 [2], it has since become distributed globally. BPIV-3 is the etiological agent of bovine parainfluenza, an acute, febrile, and contagious respiratory infection that impairs the health of cattle herds [3]. BPIV-3 is typically characterized by a 100% infection rate, respiratory impairment, and immune suppression. These factors can precipitate co-infections and secondary infections [4–7]. Notably, BPIV-3 is also a major contributor to the bovine respiratory disease (BRD) complex [8], a multifactorial disorder often accompanied by co-infections with other viral and bacterial pathogens. This condition leads to severe bronchopneumonia, reduced productivity, and increased veterinary costs [3,9,10], which in turn elevate mortality rates – averaging 10% but potentially reaching as high as 50% [3,9–12].

While inactivated vaccines targeting BPIV-3, either alone or in combination with other BRD pathogens, are commercially available, their efficacy is constrained by the lack of specific preventive targets and the complexity of co-infection involving multiple pathogens. Furthermore, there is a shortage of specific targets for the development of effective chemical therapeutics. The limited understanding of host factors crucial for BPIV-3 adsorption, invasion, and replication has partially impeded the identification of potential specific targets for developing effective control strategies. Consequently, elucidating the interactions between BPIV-3 and host cells is crucial for comprehensively understanding the host factors that govern the virus’s pathogenesis.

Currently, various methodological approaches have been employed to clarify the molecular mechanisms governing interactions between BPIV-3 and its hosts. For instance, quantitative proteomics analysis revealed that BPIV-3 infection activates the host’s p38 MAPK signaling pathway, thereby in turn facilitating viral replication [13]. RNA-seq technology has also been employed to decipher the transcriptional response of Madin-Darby bovine kidney (MDBK) cells to BPIV-3 infection of caprine origin [14]. In contrast, high-throughput genome-wide screening of CRISPR/Cas-constructed gene-deleted cell mutant libraries enables more effective identification of individual host gene functions. This approach not only enhances understanding of viral pathogenesis but also reveals novel targets for developing more effective antiviral strategies [15]. For example, genome-wide CRISPR screening of human and porcine cells has identified multiple key host factors that promote infections by viruses such as HIV [16], Influenza A virus [17], SARS-CoV-2 [18], Japanese encephalitis virus [19], Transmissible gastroenteritis virus [20], and Porcine epidemic diarrhea virus [21]. The application of CRISPR/Cas technology in bovine pathogens is also noteworthy [22]. It has facilitated the identification of the PVRL2 gene, which is essential gene for infectious bovine rhinotracheitis virus [23], and the generation of bovine tuberculosis-resistant cattle via knockout of the host NRAMP1 gene [24]. However, no studies have yet applied CRISPR/Cas systems to identify BPIV-3 host factors specific to BPIV-3. BPIV-3 is classified into three genotypes (BPIV-3a, BPIV-3b, and BPIV-3c [25]), with BPIV-3a and BPIV-3c being the most prevalent genotypes and major causes of BRD in China. This critical void highlights the need to elucidate the molecular mechanisms underlying BPIV-3a infection, which will in turn support the development of novel preventive and therapeutic strategies.

To decipher the molecular interactions between BPIV-3a and host factors, this study adopted a genome-scale CRISPR/Cas9 knockout (KO) screening approach to systematically KO genes in bovine host cells. The resulting mutant MDBK cell library was then challenged with BPIV-3a to identify virus-resistant cells. Through high-throughput sequencing, we identified novel candidate genes that potentially regulate BPIV-3a infection and validated these candidates by generating single-cell-derived KO clones. Our findings revealed that the host genes Wnt family member 5A (WNT5A), solute carrier family 16 member 13 (SLC16A13), and selenoprotein N (SELENON) are critical for the replication of BPIV-3a and BPIV-3c, and other RNA viruses such as Bovine enterovirus (BEV). These insights help address critical voids in bovine functional genomics related to virus infection.

## Materials and methods

### Construction of a bovine genome-scale CRISPR/Cas9 KO (BovGecKO) cell pool

Lenti-Cas9-Blast (Addgene, Watertown, MA, USA, Cat. No.: 53,962), pTRIP-MCS-3Flag-IRES-RFP, and pKLV2-U6gRNA5(*Bbs*I)-PGKpuro2ABFP (Addgene, Cat. No.: 67,991) were kindly provided by Professor Shuhong Zhao (College of Animal Science and Technology of Huazhong Agricultural University). Using Lenti-Cas9-Blast vector, we generated a Cas9-expression cell line (MDBK-Cas9) via lentiviral packaging.

The sgRNA library sequences used in this study were previously designed by Peng et al. (a labmate in our team, BioProject ID: PRJCA048999). For this study, bovine whole-genome data were mainly sourced from the Ensembl database, supplemented by the NCBI database, using the data version Bos_taurus.UMD3.1.cds.all. After removing duplicate genes, a total of 23,265 complete protein-coding genes were integrated. The bovine sgRNA library was designed with CRISPR-Offinder, generating 85,373 sgRNAs. The design criteria were as follows: PAM sequence at the target gene site was NGG; sgRNA length was 20 bp; GC content was 40%–60%; target site was close to the start codon ATG; each sgRNA had at least five mismatches with other sites to ensure specificity and knockout efficiency; maximum four sgRNAs were selected per gene. Additionally, 500 sgRNAs with no targets in the bovine genome were designed as negative controls, giving a total of 85,873 sgRNAs.

To generate lentivirus for the sgRNA library, we co-transfected 12 μg of the library plasmid, 4 μg of pMD2.G (Addgene, Cat. No.: 12,259), and 8 μg of psPAX2 (Addgene, Cat. No.: 12,260) into each 100-mm dish using JetPRIME (Polyplus, Strasbourg, France, Cat. No.: B180306). At 60 h (hour) post-transfection, the cell supernatants were collected and filtered through a 0.45 μm low protein-binding membrane (Millipore, Billerica, MA, USA). The lentiviral supernatant and concentrate were then mixed at a volume ratio of 4:1 (Biodragon, Beijing, China, Cat. No.: C2901S). After mixing, the solution was left to stand at 4°C, with gentle mixing every 30 min (min) for three cycles. The solution was then incubated at 4°C overnight. The mixture was centrifuged at 4,000 × g and 4°C for 30 min. The virus pellets were resuspended in phosphate-buffered saline (PBS, 0.01 M, pH 7.4), aliquoted, and stored at −80°C.

Approximately 1.4 × 10^8^ MDBK-Cas9 cells at 50% confluency were seeded into T225 flasks. After 12 h, the cells were infected with concentrated lentiviruses at a multiplicity of infection (MOI) of 0.3 [26], with polybrene added to the medium at a final concentration of 10 μg/mL (Millipore, Cat. No.: 107,689-10 G). At 24 hour post-infection (hpi), the cell supernatants were removed and replaced with fresh medium. At 2 day post-infection (dpi), the medium was replaced with fresh medium containing 2 μg/mL puromycin (InvivoGen, Toulouse, France, Cat. No.: ant-pr-1). After 5 d of drug selection, a BovGeCKO cell pool was generated. Approximately 1 × 10^7^ BovGeCKO cells were used for genomic DNA extraction with the Blood & Cell Culture DNA & Tissue Genome DNA Kit (TIANGEN, Beijing, China, Cat. No.: DP304-02) and the extracted DNA was amplified for high-throughput sequencing. All primers are listed in Supplementary Table 1.

### Illumina sequencing

The sgRNA-targeting region was amplified by PCR using Q5® Hot Start High-Fidelity DNA Polymerase (New England Biolabs, Ipswich, MA, USA, Cat. No.: M0494S), with genomic DNA from mutant cells and plasmid DNA serving as templates. PCR products were purified using a MinElute PCR Purification Kit (QIAGEN, Hilden, Germany, Cat. No.: 28,004). The purified PCR products were then further amplified by PCR using different barcoded primers. All PCR products were mixed, purified again with a MinElute PCR Purification Kit, and subjected to HiSeq 3000 next-generation sequencing.

Mapped read counts were subsequently input into the MAGeCK analysis software package (version 0.5) [27]. We conducted multiple rounds of CRISPR screening. Therefore, the top 100 genes ranked by fold change (FC) and read abundance from the 3rd and 4th rounds of BPIV-3a challenge were used as input for analysis via the DAVID Bioinformatics Resources (https://david.ncifcrf.gov/) [28], which enabled Kyoto Encyclopedia of Genes and Genomes (KEGG) enrichment and Gene Ontology (GO) analyses. The statistical results of raw reads and normalized reads of sgRNAs enriched in the 4th round of BPIV-3a screening, as well as information on the top 100 genes and their corresponding sgRNA sequences, are detailed in Supplementary Table 2.

### Cells and virus

MDBK cells (ATCC, Manassas, VA, USA, CCL-22) and HEK293T cells (ATCC, CRL-3216) were cultured in Dulbecco’s Modified Eagle’s Medium (DMEM, GIBCO BRL, Grand Island, NY, USA, Cat. No.: 12,100,046) supplemented with 10% fetal bovine serum (FBS, GIBCO BRL, Cat. No.: 16,000–044) and 1% penicillin/streptomycin (GIBCO BRL, Cat. No.: 15,140–122) at 37°C with 5% CO_2_. Cells were transfected with JetPRIME following the manufacturer’s instructions. BPIV-3a, BPIV-3c, and BEV were from our laboratory; they were isolated from clinical specimens as reported previously [29].

### BovGecKO cells challenged with BPIV-3a

Approximately 1 × 10^7^ BovGeCKO cells were seeded into T225 cell culture flasks and maintained in complete medium until 90% confluency was achieved. At this point, the cells were infected with BPIV-3a at an MOI of 0.005. Following 2 h of incubation at 37°C with 5% CO_2_, the virus inoculum was aspirated, and the cells were rinsed once with phosphate-buffered saline (PBS) before being replenished with fresh DMEM containing 2% FBS and 1% penicillin/streptomycin. At 15 dpi, the surviving cells were subcultured and expanded to sufficient quantities for high-throughput sequencing and the subsequent round of BPIV-3a infection.

### Construction of CRISPR/Cas9-mediated candidate gene KO and corresponding rescue MDBK cell lines

Paired sgRNA oligonucleotides (50 μM) targeting candidate genes were annealed (95°C for 10 min, 65°C for 60 min) and cloned into *Bbs*I-digested pKLV2-U6gRNA5-PGKpuro2ABFP (Addgene, Cat. No.: 67,991), followed by lentivirus production. sgRNA lentiviruses were transduced into MDBK-Cas9 cells, and 2 d later, medium was replaced with 2 μg/mL puromycin-containing medium. After 5 d of selection, surviving cells were single-cell seeded into 96-well plates via limiting dilution. Following 15 d of culture, genomic DNA from monoclonals was PCR-amplified, and sequencing of amplicons confirmed candidate gene KO cell lines. Primer and sgRNA sequences are in Supplementary Table 1.

For gene rescue experiments, the pTRIP-MCS-3Flag-IRES-RFP vector was digested with *Spe*I and *Sal*I (New England Biolabs, Ipswich, MA, USA, Cat. No.: R3133S, R0138S) at 37°C for 2 h, and the digestion products were verified by 1% agarose gel electrophoresis to confirm linearization. The coding sequences (CDSs) of WNT5A, SELENON, and SLC16A13 were amplified from bovine cDNA templates and inserted into the linearized vector via homologous recombination (using the ClonExpress® II One Step Cloning Kit, Vazyme, Nanjing, China, Cat. No.: C112). Synonymous codon substitutions were introduced into the sgRNA-targeted regions of each CDS to preserve the native protein sequence. All constructed plasmids were verified by Sanger sequencing (Tsingke, Beijing, China), using the primer sequences provided in Supplementary Table 1. Lentiviruses were then produced by co-transfecting the recombinant plasmids with packaging vectors (pMD2.G and psPAX2) and transduced into the corresponding KO cell lines at an MOI of 3. At 24 h post-transduction, the medium was replaced with fresh DMEM supplemented with 400 μg/mL neomycin (Beyotime, Shanghai, China, Cat. No.: ST081). Following 7 d of antibiotic selection, the rescued cell lines were successfully established.

### Reverse transcription quantitative real-time PCR (RT-qPCR)

For relative RT-qPCR, total RNA was extracted from cells using RNA-Solv® Reagent (Omega Bio-Tek, Norcross, GA, USA, Cat. No.: R6830-02), while viral nucleic acids (DNA/RNA) were extracted from cell suspensions using the EasyPure® Viral DNA/RNA Kit (TransGen, Beijing, China, Cat. No.: ER201-01), both according to the manufacturers’ instructions. RNA quantity and quality were assessed using a NanoDrop 2000 spectrophotometer (Thermo Fisher Scientific, Waltham, MA, USA), with A260/A280 ratios of 1.8–2.0 indicating high-quality RNA.

Complementary DNA (cDNA) was synthesized using HiScript III All-in-One RT SuperMix Perfect for qPCR (Vazyme, Nanjing, China, Cat. No.: R333-01) and used as the template for relative RT-qPCR. The RT-qPCR reaction was prepared in a total volume of 20 μL, containing 1 μL of cDNA and 4 μM primer pairs, with ChamQ Universal SYBR qPCR Master Mix (Vazyme, Nanjing, China, Cat. No.: Q111-02) as the detection reagent. Reactions were run on a CFX96 Real-Time PCR Detection System (Bio-Rad, Hercules, CA, USA) with the following program: 1 cycle of 95°C for 30 s (s), followed by 39 cycles of 95°C for 10 s and 60°C for 30 s. Relative gene expression levels were calculated using the 2^−∆∆Ct^ method, with glyceraldehyde-3-phosphate dehydrogenase (GAPDH) serving as the internal normalization control.

For absolute RT-qPCR, approximately 1 μL of viral RNA was used as the template for cDNA synthesis. Assays were performed in a 20 μL final reaction volume using ChamQ Universal SYBR qPCR Master Mix and specific primers targeting the nucleoprotein (NP) gene of BPIV-3a. To generate a standard curve for quantification, the cDNA of the BPIV-3a NP gene fragment was amplified, cloned into the pMD19-T vector, and used as the reference standard to determine BPIV-3a genome copy numbers. All primers used for absolute RT-qPCR are listed in Supplementary Table 1.

### Virus plaque observation and titration

Virus plaque assays were performed using MDBK cells. Briefly, wild-type (WT) and mutant MDBK cells were seeded in 12-well plates and infected with BPIV-3a at an MOI of 0.1, followed by incubation at 37°C with 5% CO_2_. After 2 h of adsorption (to allow viral attachment to cells), the culture medium was aspirated and replaced with an overlay medium consisting of 50% 2× DMEM (GENOM, Hangzhou, China, Cat. No.: GNM12802), 50% low-melting-point agarose (Beyotime, Shanghai, China, Cat. No.: Y260282, melted and cooled to 40–45°C before use), 2% FBS, and 1% penicillin/streptomycin. Plates were then incubated at 37°C with 5% CO_2_ for 3 d to allow plaque formation.

Subsequently, cells were fixed with 10% neutral buffered formaldehyde (NBF) overnight at room temperature. After removing the fixative, cells were stained with 0.5% crystal violet solution for 2 h at room temperature. Excess stain was rinsed off with distilled water, and plaques were visualized once the plates were air-dried. Images of the stained plaques were captured using a smartphone.

For virus titration, mutant and control MDBK cells (grown to ~90% confluency in 6-well plates) were infected with BPIV-3a at an MOI of 0.1. At 48–72 hpi, cells and supernatants were harvested together and subjected to three freeze-thaw cycles (−80°C to 37°C) to release intracellular viruses. Viral titers, expressed as 50% tissue culture infective dose per milliliter (TCID_50_/mL), were calculated using the Spearman-Karber method. For each viral dilution, eight replicate wells were used, and the assay was independently repeated three times to ensure reliability.

### Viral attachment and internalization assays

For the viral attachment assay, MDBK cells (including the corresponding KO cells) were incubated with BPIV-3a (MOI = 1) for 1 h at 4°C. Virus solution was removed, and the cells were washed three times with PBS (0.01 M, pH 3.0) to remove unbound viruses [30]. Then, the cells were lysed with TRIzol (Invitrogen, Cat. No.: 15,596,026) to extract total RNA. Attached viruses were detected by RT-qPCR using BPIV-3a N gene primers (Supplementary Table 1).

For viral internalization assays, MDBK cells (or corresponding KO cells) were incubated with BPIV-3a (MOI = 1) for 1 h at 4°C, washed three times with ice-cold PBS (0.01 M, pH 3.0) to remove unbound virus, then incubated with pre-warmed DMEM +2% FBS for 1 h at 37°C with 5% CO_2_. Cells were lysed with TRIzol (Invitrogen, Cat. No.: 15,596,026) to extract total RNA, and internalized virus was also detected by RT-qPCR using BPIV-3a N gene primers (Supplementary Table 1). Assays were independently repeated three times.

### Cell counting Kit-8 (CCK-8) cell viability and EdU cell proliferation assays

The cell viability assay was conducted using the CCK-8 kit (Beyotime, Shanghai, China, Cat. No.: C0037) in accordance with the manufacturer’s instructions. WT and mutant MDBK cells were seeded into 96-well flat-bottom cell culture plates at an approximate density of 2 × 10^3^ cells per well in 100 μL of DMEM. After incubation for 12, 24, and 36 h at 37°C in a 5% CO_2_ atmosphere, cell viability was assessed by adding 10 μL of CCK-8 reagent to each well, followed by a 2-h incubation at 37°C. The absorbance of each well was measured at 450 nm using a microplate reader.

For the cell proliferation assay, approximately 6 × 10^5^ WT and mutant MDBK cells were seeded per well into 6-well plates in 2 mL of complete DMEM (supplemented with 10% FBS and 1% penicillin/streptomycin). Cells were first incubated at 37°C with 5% CO_2_ for 2 h, then cell proliferation was measured using the BeyoClick™ EdU Cell Proliferation Kit with Alexa Fluor 555 (Beyotime, Shanghai, China, Cat. No.: C0075S) according to the manufacturer’s protocol. After EdU staining, cell nuclei were counterstained with 500 μL of 4,’6-diamidino-2-phenylindole (DAPI) at room temperature for 10 min in the dark.

Stained cells were observed and photographed using a fluorescence microscope. The proportion of EdU-positive cells among all cells was quantified using ImageJ software (National Institutes of Health, Bethesda, MD, USA). For each cell group, three independent wells were analyzed: three random fields were captured per well, and the assay was independently repeated three times to ensure reproducibility.

### Western blotting assay

At 24 h post-viral infection, WT and mutant MDBK cells (approximately 2 × 10^6^ cells) were harvested from 6-well plates and lysed in ice-cold immunoprecipitation (IP) lysis buffer (Beyotime, Shanghai, China, Cat. No.: P0013B) for 30 min on ice to extract total cellular proteins. Subsequently, the lysates were centrifuged at 15,871 × g for 20 min at 4°C to remove cellular debris.

Proteins in the supernatant were separated by 10% sodium dodecyl sulfate-polyacrylamide gel electrophoresis (SDS-PAGE) and transferred onto polyvinylidene fluoride (PVDF) membranes (Millipore, Bedford, MA, USA). The membranes were blocked with 5% skim milk powder (Biosharp, Guangzhou, China) in 0.01 M phosphate-buffered saline (PBS; pH 7.4) containing 0.1% Tween-20 (PBS-T) at room temperature for 3 h.

Following blocking, the membranes were incubated overnight at 4°C with primary antibodies: a laboratory-generated mouse anti-BPIV-3 antibody (against the NP protein, 1:1000 dilution), a rabbit anti-WNT5A antibody (Affinity Biosciences, Ohio, USA, Cat. No.: DF6856), and a rabbit anti-β-actin antibody (Proteintech, Rosemont, IL, USA, Cat. No.: 60,004–1-Ig) as the internal loading control. All primary antibodies were diluted according to the manufacturers’ instructions [31].

After three washes with Tris-buffered saline containing 0.1% Tween-20 (TBST; Biosharp), the membranes were incubated at room temperature for 1 h with horseradish peroxidase (HRP)-conjugated secondary antibodies: goat anti-rabbit IgG (Abbkine, Wuhan, China, Cat. No.: A21020) or goat anti-mouse IgG (Abbkine, Wuhan, China, Cat. No.: A21010). Finally, protein bands were visualized using an enhanced chemiluminescence (ECL) detection system (Bio-Rad, Hercules, CA, USA, Cat. No.: 170–5061).

### Transfection of siRNAs

WNT5A small interfering RNA (siRNA) sense strand (+): 5’-GGCUACGACCAGUUCAAGA (dT)(dT)-3,’ antisense strand (-): 5’-UCUUGAACUGGUCGUAGCC (dT)(dT)-3.’ All siRNAs were synthesized by TsingKe (Beijing, China).

Cells were plated into 6-well plates and grown to approximately 60% confluency, then transfected with 110 pmol of the respective siRNAs using Lipofectamine 2000 (Invitrogen, Carlsbad, CA, USA, Cat. No.: 11,668,500) according to the manufacturer’s instructions. For the negative control, cells were transfected with non-targeting control siRNA (siRNA-NC). At 12 h post-transfection, cells were infected with BPIV-3a at an MOI of 0.1. At 12, 18, 24, 30, and 36 hpi, the infected cells were collected for subsequent analyses.

### Statistical analysis

For all experiments, each treatment group was set up in triplicate wells, and each independent experiment was repeated three times unless otherwise specified to ensure reproducibility. Experimental data are presented as the mean ± standard error of the mean (SEM) derived from three biological replicates. Statistical analyses were performed using GraphPad Prism software (Version 9.5.0; GraphPad, Boston, MA, USA). A two-tailed Student’s *t*-test was used to compare differences between two groups, and one-way analysis of variance (ANOVA) followed by Tukey’s post-hoc test was employed for multiple group comparisons. Statistical significance was defined as follows: *p*-value < 0.05 (*), < 0.01 (**), < 0.001 (***), and < 0.0001 (****); *p*-value ≥0.05 was considered non-significant.

## Results

### Genome-scale CRISPR/Cas9 KO screen for host factors essential for BPIV-3 replication in MDBK cells

To identify host factors involved in BPIV-3 replication, we performed a BovGeCKO screening in MDBK-Cas9 cells. Analysis showed that the mutant cell library covered 92.12% of the originally designed sgRNA sequences and targeted 99.62% of the protein-coding genes in the bovine genome (Experiment accession ID: CRX2077381). Additionally, a sequencing coverage of >500-fold per sgRNA was maintained throughout the loss-of-function screening (Supplementary Figure S1A). Both the BovGeCKO sgRNA plasmid library and the mutant cell library contained four sgRNAs per gene for most protein-coding genes (Supplementary Figure S1B). Thus, a highly active and specific mutant cell library was successfully constructed, which is ideal for genome-wide loss-of-function screening.

**Figure 1.**
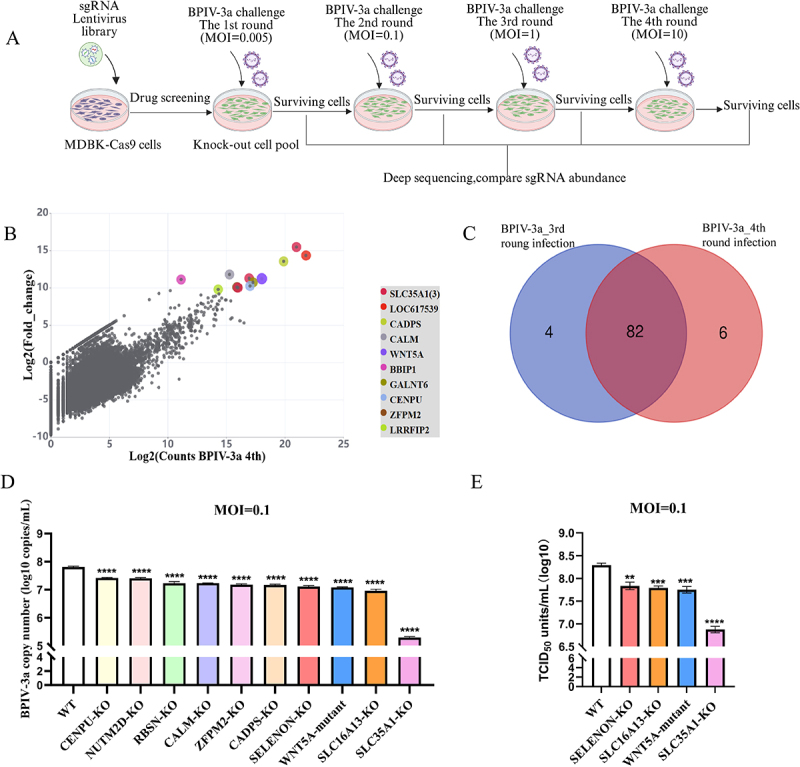
Genome-scale CRISPR screen strategy to identify host factors for BPIV-3a replication in MDBK cells.

Next, we used this loss-of-function screen with our established MDBK mutant cell library to identify host genes for BPIV-3a infection, with our established MDBK mutant cell library. Initially, we tested WT MDBK cells infected with BPIV-3a at MOIs of 0.0005, 0.005, 0.05, and 0.5 to assess how inoculation efficiency correlates with cell death. At an MOI of 0.005, only minimal CPEs were detected at 1 dpi; these progressed to extensive pathological changes (syncytia, cell shrinkage, and detachment) by 3 dpi and finally resulted in complete cell death (Supplementary Figure S2). We then performed four BPIV-3a challenge rounds, with MDBK-Cas9 cells as negative control to confirm infection-induced death. In round 1 (MOI = 0.005), all infected control cells died, while BovGeCKO mutant cells survived. Survivors were screened at higher MOIs (0.1, 1, 10; Figure 1(A)). Genomic DNA from survivors of rounds 3–4 was used for PCR and high-throughput sequencing.

(A). Schematic presenting the generation of the BovGeCKO cell library with four subsequent rounds of BPIV-3a challenge. (B). Scatter plot presenting the sgRNA enrichment profiles after the fourth round of BPIV-3a challenge compared with that of the untreated mutant cell library. (C). Venn diagram demonstrating overlapping enrichment of the top 100 sgRNAs from the third and fourth rounds of BPIV-3a challenge. (D). Comparison of BPIV-3a copy numbers after infection with 0.1 MOI in 10 candidate gene KO cell lines versus WT MDBK cells. (E). TCID_50_ of the KO cell lines of the candidate genes WNT5A, SELENON, and SLC16A13 after infection with BPIV-3a at an MOI of 0.1. For D and E, the experimental results were based on three technical replicates. ANOVA was used to analyze statistical differences among the groups. **p < 0.01; ***p < 0.001; ****p < 0.0001.

The CRISPR screening results demonstrated that, after the third round of BPIV-3a challenge, the top 10 candidate genes with the highest enrichment (in decreasing order) were SLC35A1, NUTM2D, CADPS, CALM, BBIP1, CENPU, RBSN, GINS1, PDE12, and GALNT6. Notably, the most highly enriched gene SLC35A1 encodes a well-characterized paramyxovirus cellular receptor and was identified in this screening (Supplementary Figure S3A). Following the fourth round of BPIV-3a challenge, the top 10 enriched candidate genes were SLC35A1, NUTM2D, CADPS, CALM, WNT5A, BBIP1, GALNT6, CENPU, ZFPM2, and LRRFIP2; the two rounds differed by only three genes (Figure 1(B), Supplementary Table 2). Furthermore, overlap analysis of sequencing data from the third and fourth rounds showed substantial overlap between the enriched genes, with 82% of the top 100 enriched genes shared between the two rounds (Figure 1(C)). The consistency of this enrichment pattern across multiple BPIV-3a challenge rounds strongly suggests that these candidate genes are likely to play critical roles in mediating the host response to BPIV-3a infection.

To categorize the top 100 enriched genes from rounds 3–4 of BPIV-3a challenge, we performed GO and KEGG enrichment analyses, showing enrichment in extracellular exosomes, metabolic pathways, and Golgi apparatus (Supplementary Figure S3B). Early one-step growth curves in MDBK-Cas9 cells (MOIs 0.1, 1.0) showed MOI = 0.1 induced 80% CPEs at 48 h (sufficient for observing KO effects), while MOI = 1.0 induced 80% CPEs at 24–30 h (too fast). Thus, MOI = 0.1 was used for validation (Supplementary Figure S3C). Based on the results of the fourth round of screening (Experiment accession ID: CRX2077382), we selected 10 genes to construct single-cell KO clones. Absolute RT-qPCR detection showed that at 24 (MOI = 0.1), the BPIV-3a copy numbers in KO cells were all reduced by more than 0.45 log_10_ compared with WT cells (Figure 1(D)). Viral titration showed WNT5A, SELENON and SLC16A13 KO cells had the largest titer reductions (*p* < 0.01; Figure 1(E)). In conclusion, our CRISPR-based loss-of-function screen successfully identified these essential genes, including the well-characterized receptor SLC35A1 and the newly discovered WNT5A, SELENON, and SLC16A13, as critical regulators of BPIV-3a replication.

### WNT5A modulated BPIV-3 replication

**Figure 2.**
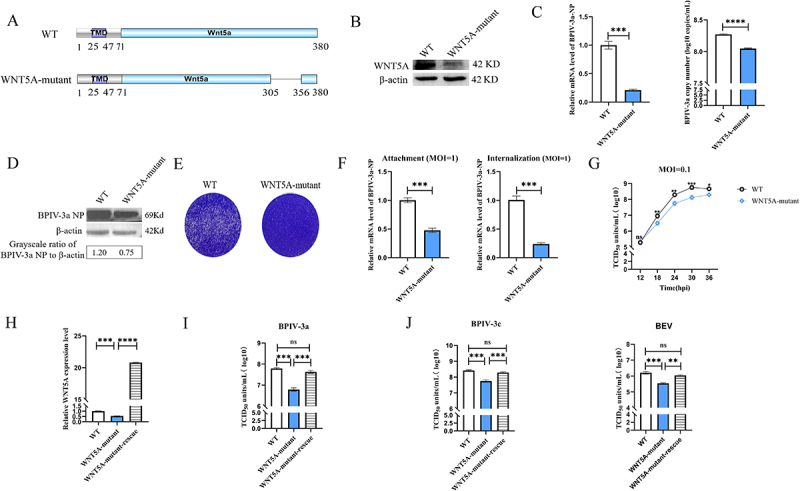
The WNT5A gene is involved in BPIV-3 replication.

To elucidate WNT5A’s role in BPIV-3 infection, we generated WNT5A-mutant cell lines using CRISPR/Cas9. Sanger sequencing confirmed two mutant clones with 150-bp and 27-bp deletions relative to the WT genome (Supplementary Figure S4). Via one-step growth curve assays, following BPIV-3a infection at MOI 0.1, mutant clones showed comparable growth dynamics, and no significant differences were detected between clones (ANOVA, *p* > 0.05; Supplementary Figure S5A). Thus, we selected WNT5A-mutant-1 (harboring a 150-bp/50-amino-acid deletion in exon 1; Figure 2(A)) for subsequent studies. Western blotting and RT-qPCR confirmed reduced WNT5A expression in this mutant, with no subsequent effects on cell proliferation or viability (Figure 2(B,H), Supplementary Figure 6). At 24 hpi with BPIV-3a (MOI 0.1), WNT5A-mutant cells showed a 5-fold reduction in BPIV-3a NP gene expression and a 0.3 log_10_ decrease in supernatant viral load, indicating significantly lower intracellular and extracellular BPIV-3a levels (Figure 2(C)). At the protein level, NP expression was 1.6-fold lower in mutants vs WT cells (Figure 2(D)).

(A). Determination of the amino acid sequences in WT and WNT5A-mutant MDBK cells. (B). Western blotting of WNT5A protein expression in WT and WNT5A-mutant MDBK cells. (C). Comparison of intracellular and extracellular BPIV-3a levels in WT and WNT5A-mutant MDBK cells. Intracellular BPIV-3a-NP gene expression was determined by relative RT-qPCR, whereas extracellular BPIV-3a viral copy numbers were determined by absolute RT-qPCR. (D). Western blotting of BPIV-3a NP expression in WT and WNT5A-mutant MDBK cells. (E). Plaque assays with crystal violet staining to observe BPIV-3a plaques in infected WT and WNT5A-mutant MDBK cells. (F). The impact of WNT5A mutation on BPIV-3a attachment and internalization in MDBK cells was evaluated using RT-qPCR. (G). One-step growth curve to determine the differences in BPIV-3a replication between WT and WNT5A-mutant MDBK cells. (H). Relative RT-qPCR to detect WNT5A gene expression in WNT5A-mutant and WNT5A-mutant-rescue MDBK cells. (I). Viral titers of BPIV-3a in WT, WNT5A-mutant, and WNT5A-mutant-rescue MDBK cells. (J). Replication of BPIV-3c and BEV in WT, WNT5A-mutant, and WNT5A-mutant-rescue MDBK cells. The experimental results were based on three technical replicates, and representative data are presented. Student’s *t*-test was applied for between-group comparisons, and ANOVA was used for multiple-group comparisons. ns, not significant; *p < 0.05; **p < 0.01; ***p < 0.001; ****p < 0.0001.

Plaque assays further demonstrated that WNT5A-mutant cells were resistant to BPIV-3a-induced cell death (Figure 2(E)). Attachment and internalization assays (BPIV-3a, MOI 1) showed that WNT5A mutation significantly inhibited both processes (Figure 2(F)). For replication kinetics, viral titers measured at 12–36 hpi (MOI 0.1) revealed that WNT5A mutation significantly suppressed BPIV-3a replication during mid-to-late stages (18–36 hpi; Figure 2(G)).

To confirm specificity, we knocked down WNT5A in WT MDBK cells using RNA interference (RNAi) technology. Consistent with data from WNT5A-mutant cells, WNT5A knockdown inhibited viral attachment, internalization, and replication (Supplementary Figure 7). Conversely, we ectopically expressed WNT5A in WNT5A-mutant cells (Figure 2(H)), which restored BPIV-3a titers to WT levels at 24 h post-infection (hpi, MOI 0.1; Figure 2(I)). Together, these results indicate that WNT5A is essential for efficient BPIV-3a replication.

Finally, we investigated the role of WNT5A in BPIV-3c and BEV infection (MOI 0.1) using WNT5A-mutant and WNT5A-rescued cells. The results showed that replication of both viruses was significantly reduced in WNT5A-mutant cells, while WNT5A-rescued cells exhibited WT-like viral titers (Figure 2(J)). This confirms WNT5A as a critical host factor for bovine viruses, including BPIV-3a, BPIV-3c, and BEV.

### Involvement of SELENON in BPIV-3 replication

**Figure 3.**
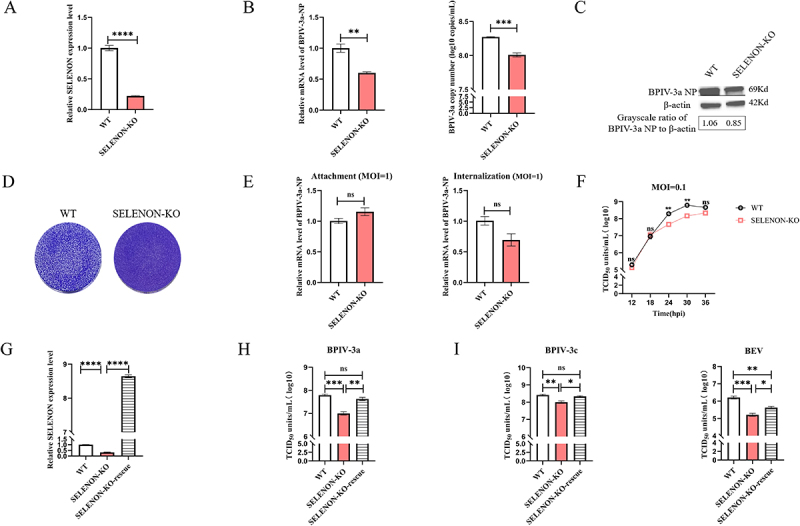
The SELENON gene is involved in the replication of BPIV-3.

SELENON-KO cells were established by introducing SELENON-targeting sgRNA into MDBK-Cas9 cells. Sanger sequencing confirmed that both selected SELENON-KO lines contained a 13-bp deletion, leading to frameshift mutations in the coding region (Supplementary Figure S4). A one-step growth curve assay, with BPIV-3a infection at MOI 0.1, showed consistent phenotypes and growth dynamics across KO clones (ANOVA, *p* > 0.05; Supplementary Figure 5B), so SELENON-KO-1 was chosen for subsequent experiments. RT-qPCR verified significant downregulation of SELENON mRNA in KO cells (Figure 3(A)), while EdU and CCK-8 assays revealed no differences in cell proliferation or viability between SELENON-KO and WT MDBK cells (Supplementary Figure S8).

(A). Relative RT-qPCR of SELENON transcription levels in WT and SELENON-KO MDBK cells. (B). Comparison of intracellular and extracellular BPIV-3a NP gene expression and BPIV-3a copy numbers in WT and SELENON-KO MDBK cells. Intracellular BPIV-3a-NP gene expression was determined using relative RT-qPCR, whereas the extracellular BPIV-3a viral copy number was assessed using absolute RT-qPCR. (C). Western blotting of the expression of BPIV-3a NP protein in infected WT and SELENON-KO MDBK cells. (D). Plaque assay with crystal violet staining to observe BPIV-3a plaques in infected WT and SELENON-KO MDBK cells. (E). The impact of SELENON-KO on BPIV-3a attachment and internalization in MDBK cells was evaluated using RT-qPCR. (F). One-step growth curve assay of BPIV-3a replication in WT and SELENON-KO MDBK cells. (G). Relative RT-qPCR of SELENON mRNA expression after ectopic expression. (H). Viral titers of BPIV-3a in WT, SELENON-KO, and SELENON-KO-rescue MDBK cells. (I). Replication of BPIV-3c and BEV in WT, SELENON-KO and SELENON-KO-rescue MDBK cells. The experimental results were based on three technical replicates, and representative results are presented. Student’s *t*-test was applied for between-group comparison, and ANOVA was used for multiple-group comparisons. ns, not significant; *p < 0.05; **p < 0.01; ***p < 0.001; ****p < 0.0001.

At 24 hpi with BPIV-3a (MOI 0.1), SELENON-KO cells showed 1.7-fold lower NP gene expression and a 0.3 log_10_ reduction in supernatant viral load compared with WT cells, indicating significantly decreased intracellular and extracellular BPIV-3a levels (Figure 3(B)). Western blotting further confirmed 1.3-fold lower NP protein expression in KO vs WT cells (Figure 3(C)). Plaque assays also demonstrated that SELENON-KO cells were significantly resistant to BPIV-3a-induced cell death (Figure 3(D)). However, attachment and internalization assays (BPIV-3a, MOI 1) showed no effect of SELENON-KO on these processes (Figure 3(E)).

To assess SELENON’s role in BPIV-3a replication, viral titers were measured at 12–36 hpi (MOI 0.1). Results showed that SELENON deficiency significantly inhibited BPIV-3a replication during late stages (Figure 3(F)). To rule out off-target effects, we ectopically expressed SELENON in SELENON KO cells; this intervention restored the cells’ susceptibility to BPIV-3a (Figure 3(G,H)). Additionally, SELENON-KO significantly inhibited replication of BPIV-3c and BEV compared with WT cells (Figure 3(I)), demonstrating that SELENON is required for the replication of BPIV-3a, BPIV-3c, and BEV.

### Involvement of SLC16A13 in BPIV-3 replication

SLC16A13, a member of the solute carrier family 16, was identified as a novel host factor that promotes BPIV-3a replication. To validate its function, we generated SLC16A13-KO cell lines using CRISPR/Cas9. Sanger sequencing revealed that, compared with WT cells, the two SLC16A13-KO monoclonal lines carried either a single cytidine insertion or a combined 13-bp insertion/9-bp deletion (Supplementary Figure S4). One-step growth curve analysis, following BPIV-3a infection at MOI 0.1, showed consistent phenotypes and growth dynamics across KO clones, with no significant inter-clone differences (ANOVA, *p* > 0.05; Supplementary Figure 5C). Thus, SLC16A13-KO-1 was selected as the representative clone for subsequent experiments.

**Figure 4.**
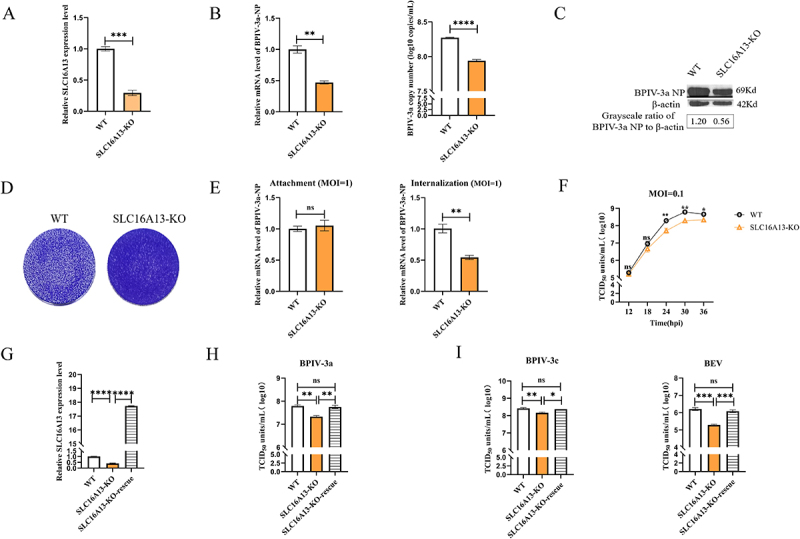
The SLC16A13 gene is involved in the replication of BPIV-3.

RT-qPCR confirmed significant downregulation of SLC16A13 transcription in KO cells (Figure 4(A)). Additionally, EdU proliferation assays and CCK-8 tests demonstrated that SLC16A13 KO had no significant effect on cell proliferation or viability (Supplementary Figure 9). At 24 hpi with BPIV-3a (MOI 0.1), culture supernatants and cells were collected, and BPIV-3a viral load and NP gene expression were quantified via RT-qPCR. Results showed that NP gene expression was 2-fold lower in SLC16A13-KO cells than in WT cells, while supernatant viral copy numbers were reduced by 0.4 log_10_ in KO cells (Figure 4(B)). Western blotting further confirmed 2.1-fold lower BPIV-3a NP protein expression in KO vs WT cells (Figure 4(C)). Collectively, these data indicate that SLC16A13 KO reduces both intracellular and extracellular BPIV-3a levels.

(A). Relative RT-qPCR of SLC16A13 transcription levels in WT and SLC16A13-KO MDBK cells. (B). RT-qPCR of intracellular and extracellular BPIV-3a NP mRNA levels and BPIV-3a copy numbers in WT and SLC16A13-KO MDBK cells. Intracellular BPIV-3a NP gene expression was determined using relative RT-qPCR, whereas extracellular BPIV-3a viral copy numbers were determined using absolute RT-qPCR. (C). Western blotting of BPIV-3a NP protein expression in WT and SLC16A13-KO MDBK cells. (D). Plaque assay with crystal violet staining to observe BPIV-3a plaques in infected WT and SLC16A13-KO MDBK cells. (E). The impact of SLC16A13-KO on BPIV-3a attachment and internalization in MDBK cells was evaluated using RT-qPCR. (F). One-step growth curve assay of BPIV-3a replication in WT and SLC16A13-KO MDBK cells. (G). Relative RT-qPCR of SLC16A13 mRNA expression after ectopic expression. (H). Viral titers of BPIV-3a in WT, SLC16A13-KO and SLC16A13-KO-rescue MDBK cells. (I). Replication of BPIV-3c and BEV in WT, SLC16A13-KO and SLC16A13-KO-rescue MDBK cells. The experimental results were based on three technical replicates, and representative results are presented. Student’s *t*-test was applied for between-group comparisons, and ANOVA was used for multiple-group comparisons. ns, not significant; *p < 0.05; **p < 0.01; ***p < 0.001; ****p < 0.0001.

Plaque assays also demonstrated that SLC16A13-KO cells were significantly resistant to BPIV-3a-induced cell death (Figure 4(D)). To assess SLC16A13’s role in BPIV-3a attachment and internalization, KO and WT cells were infected with BPIV-3a at MOI 1. RT-qPCR revealed that while BPIV-3a attachment to KO cells was unaffected, viral internalization was significantly inhibited compared with WT cells (Figure 4(E)). We then investigated SLC16A13’s impact on the BPIV-3a replication cycle: measuring viral titers at multiple time points post-infection (MOI 0.1) showed that SLC16A13 KO affected the late stage of BPIV-3a replication (Figure 4(F)).

To rule out off-target effects, a rescue experiment was performed by ectopically expressing the SLC16A13 protein-coding region (Figure 4(G)), and rescued cells restored BPIV-3a-induced cell death (Figure 4(H)). To further explore the antiviral potential of SLC16A13 KO, we examined its role in BPIV-3c and BEV replication. TCID_50_ assays confirmed that SLC16A13 is critically involved in the replication of BPIV-3a, BPIV-3c, and BEV (Figure 4(I)). Together, these results demonstrate that SLC16A13 is required for the replication of BPIV-3 (including BPIV-3a and BPIV-3c) and BEV.

## Discussion

Both BPIV-3a and BPIV-3c are common pathogens associated with BRD; they can also exacerbate BRD when co-infected with other BRD-causing pathogens [1,8]. Current control strategies primarily rely on inactivated vaccines, which have room for improvement. Identifying host factors critical for BPIV-3 infection is therefore valuable for developing novel vaccines and therapeutics to better control BPIV-3 spread. In this study, we performed BovGeCKO screening in MDBK cells and identified multiple previously unrecognized host factors that are required for BPIV-3a replication. We further confirmed that WNT5A, SELENON, and SLC16A13 are key host factors involved in infection by BPIV-3a, as well as by BPIV-3c and other bovine RNA viruses (e.g. BEV). To our knowledge, this is the first study to use BovGeCKO screening and identify host factors associated with BPIV-3.

While genome-wide CRISPR screens are widely recognized as effective tools for discovering novel host factors in viral and bacterial infections, library quality – including sgRNA coverage and screening strategies – critically impacts the reliability of results. To ensure comprehensive genome-wide target coverage, we constructed the BovGeCKO library in MDBK cells, using an sgRNA library that targets 23,265 bovine protein-coding genes. Validation results showed the mutant library covered 92.12% of the originally designed sgRNA sequences and targeted 99.62% of the protein-coding genes, confirming its robustness.

We subjected the BovGeCKO library to four rounds of BPIV-3a challenge with progressively increasing MOIs (0.005, 0.1, 1, 10). This approach not only enhanced gene-editing efficiency and optimized screening outcomes but also improved reliability and minimized false negatives. Consistently, sequencing results after the third and fourth challenge rounds revealed 82% overlap among the top 100 enriched genes. Among the top 10 enriched genes, three sgRNAs specifically targeted SLC35A1, a well-characterized Paramyxoviridae receptor [32,33]. This aligns with previous CRISPR/Cas9 knockout screening in human A549 cells, which also identified SLC35A1 as critical for paramyxovirus infection [34], further validating the reliability of our screening results.

Additionally, we used infected cell survival as the phenotype for loss-of-function screening to identify genes involved in viral adsorption, invasion, and replication. We successfully identified and validated 10 host genes that significantly promote BPIV-3 infection efficiency. Beyond the top-ranked SLC35A1, the candidate genes included NUTM2D, CADPS, CALM, WNT5A, CENPU, ZFPM2, RBSN, SELENON, and SLC16A13. We further characterized the roles of WNT5A, SELENON, and SLC16A13 in BPIV-3 infection, confirming their involvement in adsorption, invasion, and/or replication.

As a cellular autocrine protein widely distributed in the extracellular space and matrix, WNT5A is a core component of the Wnt signaling pathway – a key regulator of cellular signaling and immune responses [35]. While direct evidence linking WNT5A to viral infection is scarce, the Wnt pathway is generally recognized as a critical modulator of replication for diverse viruses [36], including human coronaviruses [37] and herpesviruses [38]. Our study is the first to demonstrate that WNT5A is essential for efficient BPIV-3 replication, with roles in viral adsorption, invasion, and replication, highlighting its potential as a target for antiviral strategies.

A multifunctional endoplasmic reticulum membrane protein, SELENON has primarily been studied in the context of antioxidant stress defense [39], calcium homeostasis regulation [40], and muscular diseases [41]. No direct evidence has linked SELENON to viral infection; however, recent studies reported differential SELENON expression in COVID-19 patients [42], suggesting a potential association with viral pathogenesis. Our findings build on this by confirming SELENON as a key host factor for BPIV-3 infection, with a specific role in viral replication (but not adsorption or invasion). The underlying mechanism requires further investigation.

Encoding a protein primarily localized to the Golgi apparatus and cytoplasm, SLC16A13 plays an important role in transmembrane transport. For instance, its encoded monocarboxylate transporter regulates cellular metabolism and maintains energy balance, while SLC16A13 polymorphisms are closely associated with diseases such as type 2 diabetes [43] and hepatitis B-related liver cancer [44], underscoring its central role in cellular metabolism and disease regulation. No reports have directly linked SLC16A13 to viral replication; however, our observation that SLC16A13 KO significantly inhibits BPIV-3 internalization and subsequent replication aligns with its transmembrane transport function. Further studies are needed to elucidate the specific mechanism by which SLC16A13 mediates BPIV-3 infection.

Notably, WNT5A, SELENON, and SLC16A13 exerted similar enhancing effects on both the single-stranded negative-sense RNA virus BPIV-3c and the single-stranded positive-sense RNA virus BEV, highlighting their broad and critical roles in facilitating viral infection. Future work will focus on uncovering the precise mechanisms by which these genes mediate viral adsorption, invasion, and replication, laying a scientific foundation for novel antiviral strategies.

In conclusion, using CRISPR-based functional genomic screening, this study identified multiple host genes required for BPIV-3a infection and confirmed that WNT5A, SELENON, and SLC16A13 are indispensable for BPIV-3a replication by promoting viral adsorption, invasion, and/or replication. These functions were also conserved in BPIV-3c and other RNA viruses. These findings provide promising targets for developing novel antiviral vaccines, therapeutics, and genetic breeding strategies against BPIV-3 and related bovine viruses.

## Supplementary Material

supplemental figure 4.tif

Revise the Supplementary figures_BPIV_3 screening_clean.docx

Supplementary Figure 3.tif

Clean Copy of Supplementary Table 1.xlsx

Supplementary Figure 9.tif

Supplementary Figure 6.tif

Supplementary Figure 8.tif

Supplementary figure1.tif

supplemental figure 7.tif

Supplementary Table 2.xlsx

Supplementary Figure 2.jpeg

supplemental figure 5.tif

## Data Availability

All data generated or analyzed in this study are publicly available in recognized repositories. Raw sequencing data can be accessed via the project ID CRA031717 in the GSA Run Selector (https://ngdc.cncb.ac.cn/gsa/browse/CRA031717). The data that support the findings of this study are openly available in Figshare at http://doi.org/10.6084/m9.figshare.28087877 [45].
