# Supplementary material for: Genome-scale CRISPR screen identifies host factors associated with bovine parainfluenza virus 3 infection
Source: Virulence. 2025 Dec 2;16(1):2589554. doi: 10.1080/21505594.2025.2589554 (PMC12674325; doi:10.1080/21505594.2025.2589554)
Supplement: Revise the Supplementary figures_BPIV_3 screening_clean.docx [file KVIR_A_2589554_SM7953.docx]

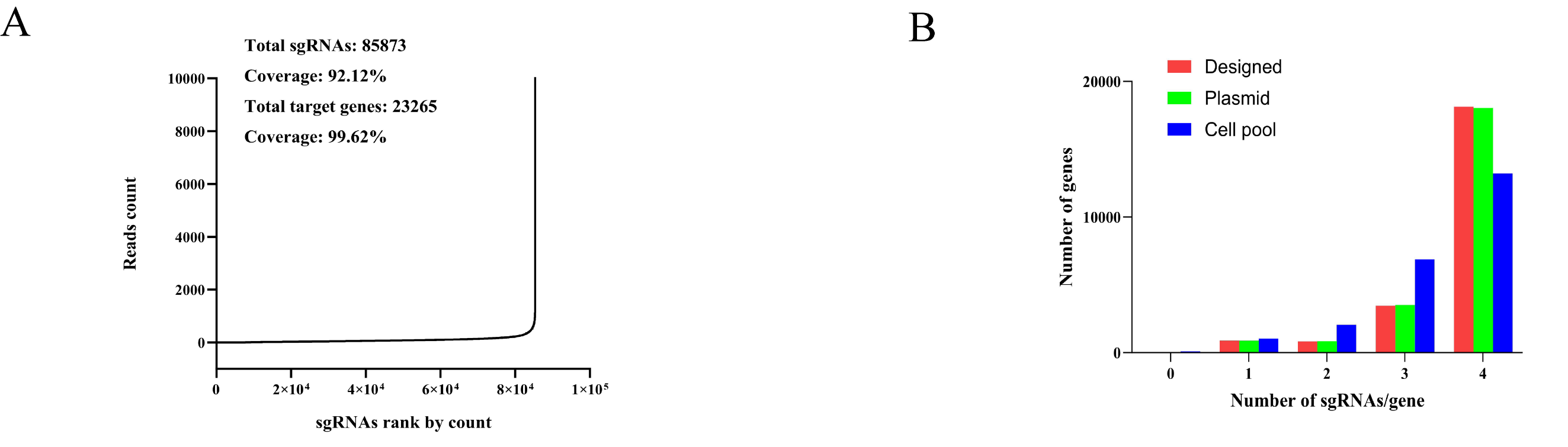


Supplementary Figure 1. Generation of a Bovine Genome-wide Cellular Mutant Library. A. Sequencing results of sgRNAs in the cellular mutant library. B. Number of sgRNAs per gene in the sgRNA library designed, plasmid library, or mutant cell pool. sgRNA, small guide RNA; Designed, the designed sgRNA library; Plasmid, sgRNA plasmid library; Cell pool, mutant cell library.


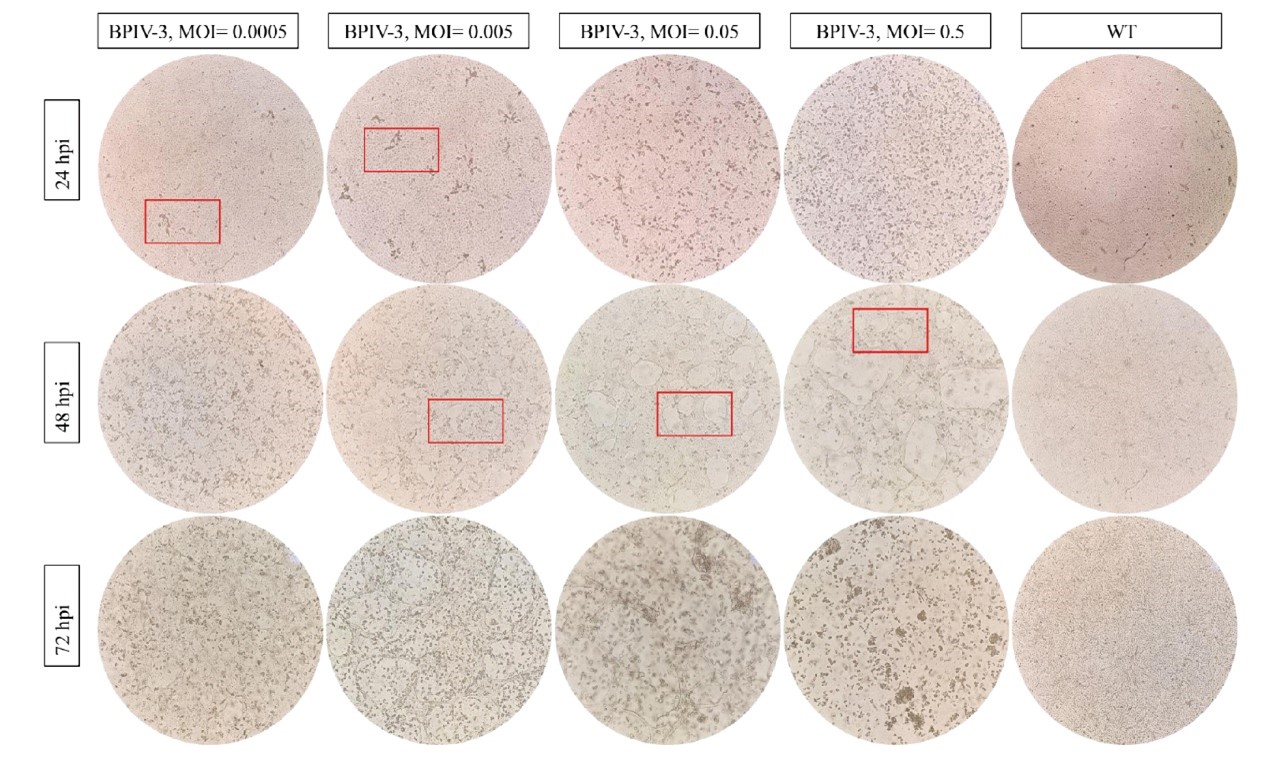


Supplementary Figure 2. Determination of the Optimal Infection Ratio of BPIV-3 in the Cellular Mutant Library. The image was captured using a smartphone under 40x magnification; the red box highlights the typical lesions caused by BPIV-3.

Supplementary Figure 3. Screening Results from the BPIV-3-Infected Cell Mutant Library. A. scatter plot representing the enrichment changes of sgRNAs in the third round of BPIV-3 challenge compared to the untreated mutant cell library. B. GO and KEGG pathway enrichment analysis results targeting the top 100 sgRNAs in both the third and fourth rounds of BPIV-3 challenges. C. One-step BPIV-3a growth curve determination.

Supplementary Figure 4. Gene Mutation Types. Red indicates sgRNA sequences; green indicates PAM sequences.


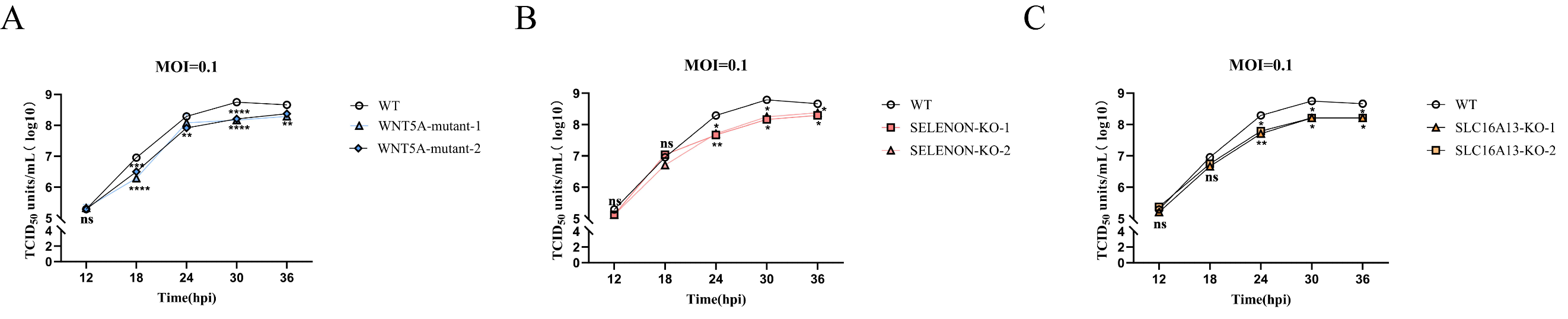


Supplementary Figure 5. One-step growth curve of BPIV-3a after infection of WT and candidate knockout cells at 0.1 MOI. A. One-step growth curve of BPIV-3a in WT and WNT5A-mutant MDBK cells. B. One-step growth curve of BPIV-3a in WT and SELENON-KO MDBK cells. C. One-step growth curve of BPIV-3a in WT and SLC16A13-KO MDBK cells. The experimental results were subjected to three technical replications, and a representative result was presented. The ANOVA was used to analyze the statistical difference among the groups. ns, non-significant; **p* < 0.05; ** *p* < 0.01; *** *p* < 0.001; *****p* < 0.0001.


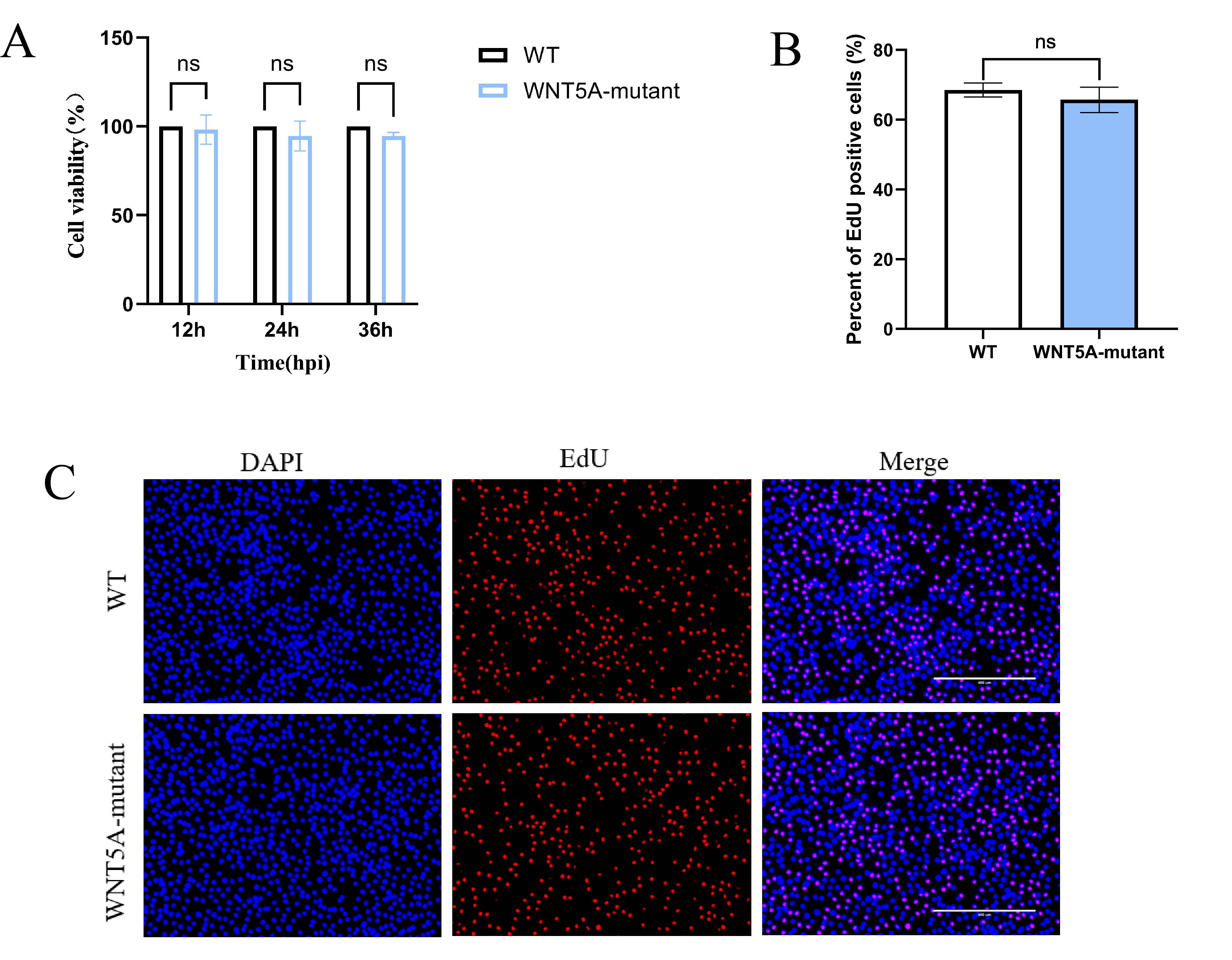


Supplementary Figure 6. Validation of Cell Proliferation Rate and Viability between WNT5A Mutant Cells and WT MDBK Cells. A. Validation of cell viability in WNT5A mutant cells and WT MDBK cells using a CCK-8 assay. B. Analysis results of EdU cell proliferation assay data. C. Detection of proliferation in WNT5A mutant cells using an EdU cell proliferation assay. The experimental results were subjected to three technical replications and the representative one of them was presented. Student’s *t*-test for a single comparison and ANOVA for more than one comparison were used. ns, non-significant.


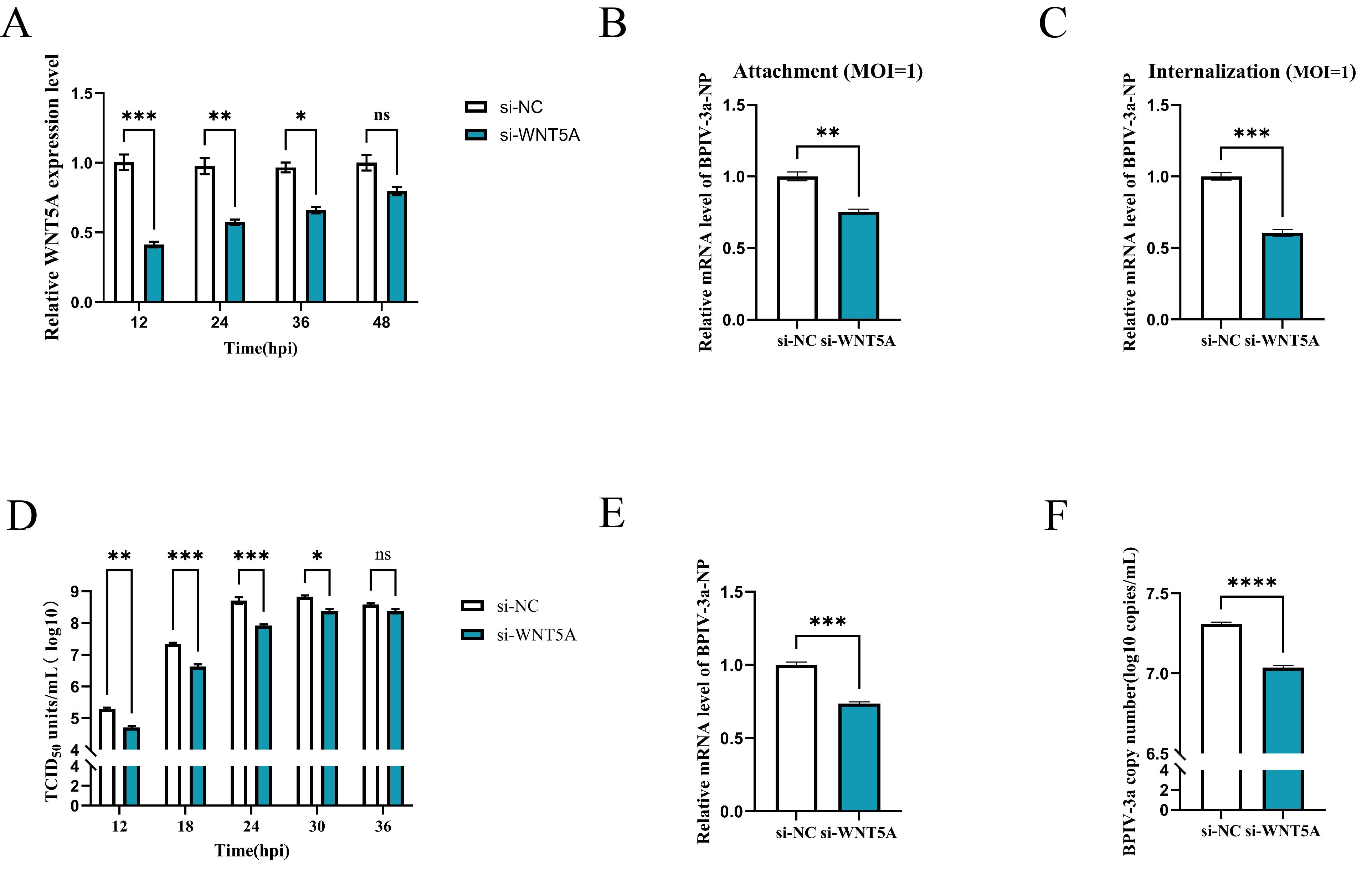


Supplementary Figure 7. Effects of knockdown WNT5A gene on BPIV-3a replication in MDBK cells. A. Relative mRNA levels of WNT5A in MDBK cells at different time points after transfection with si-WNT5A or si-NC. B-C. Effects of WNT5A knockdown on BPIV-3a attachment and internalization in MDBK cells, as assessed by RT-qPCR at 12 h post-transfection (1 MOI BPIV-3a infection). D. Viral titers of BPIV-3a in culture supernatants collected at indicated time points after infection (0.1 MOI BPIV-3a at 12 h post-transfection). E-F. Intracellular and extracellular BPIV-3a levels at 24 hpi (0.1 MOI). Intracellular BPIV-3a-NP gene expression was determined by relative RT-qPCR, whereas extracellular BPIV-3a viral copy numbers were determined by absolute RT-qPCR. The experimental results were based on three technical replicates, and representative data are presented. Student’s *t*-test was applied for between-group comparisons, and ANOVA was used for multiple-group comparisons. * *p* < 0.05; ** *p* < 0.01; *** *p* < 0.001; *****p* < 0.0001. ns, non-significant.


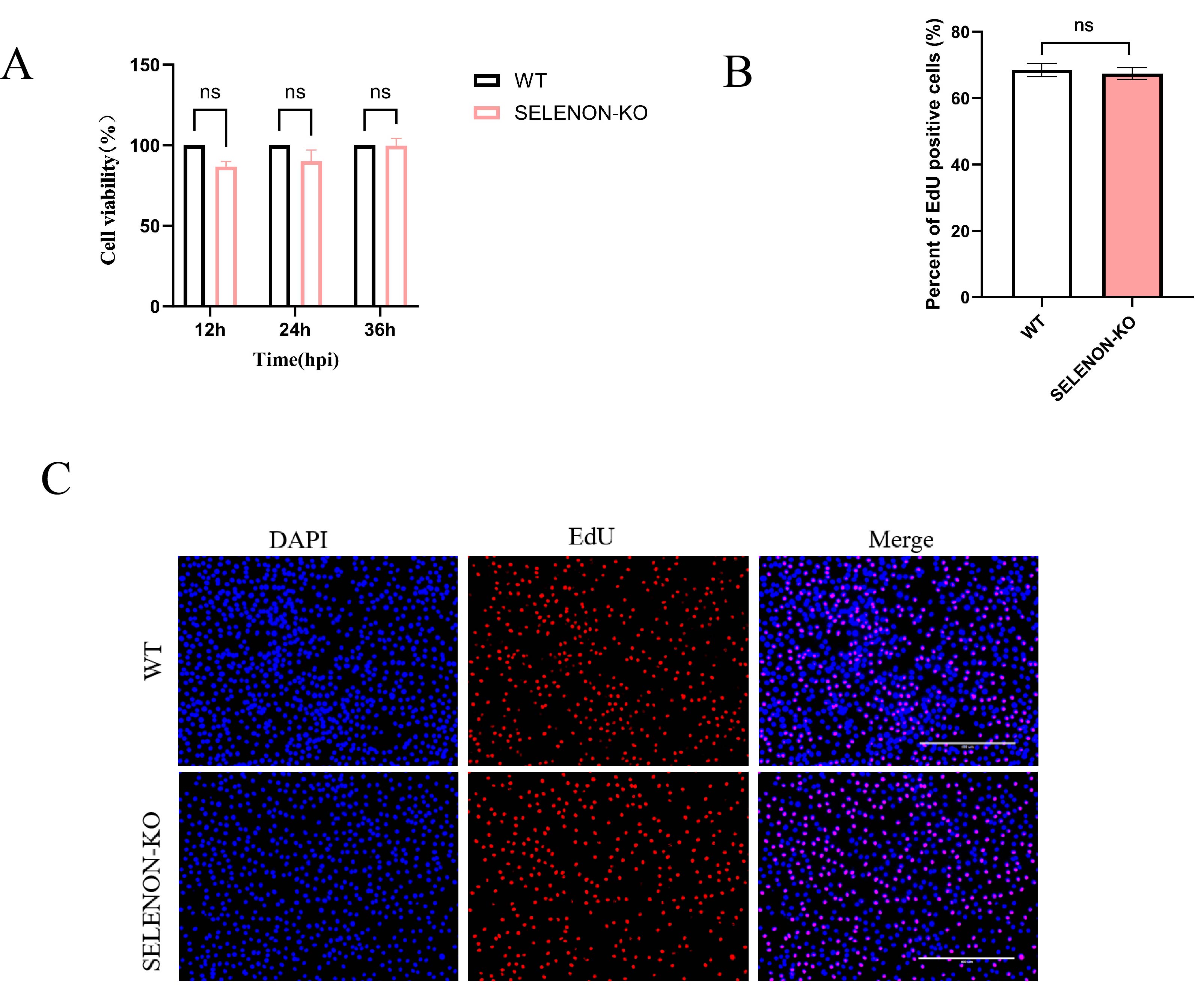


Supplementary Figure 8. Validation of Cell Proliferation Rate and Viability between SELENON KO Cells and WT MDBK Cells. A. Validation of cell viability in SELENON KO cells and WT MDBK cells using a CCK-8 assay. B. Analysis results of EdU cell proliferation assay data. C. Detection of proliferation in SELENON KO cells using an EdU cell proliferation assay. The experimental results were subjected to three technical replications and the representative one of them was presented. Student’s *t*-test for a single comparison and ANOVA for more than one comparison were used. ns, non-significant.


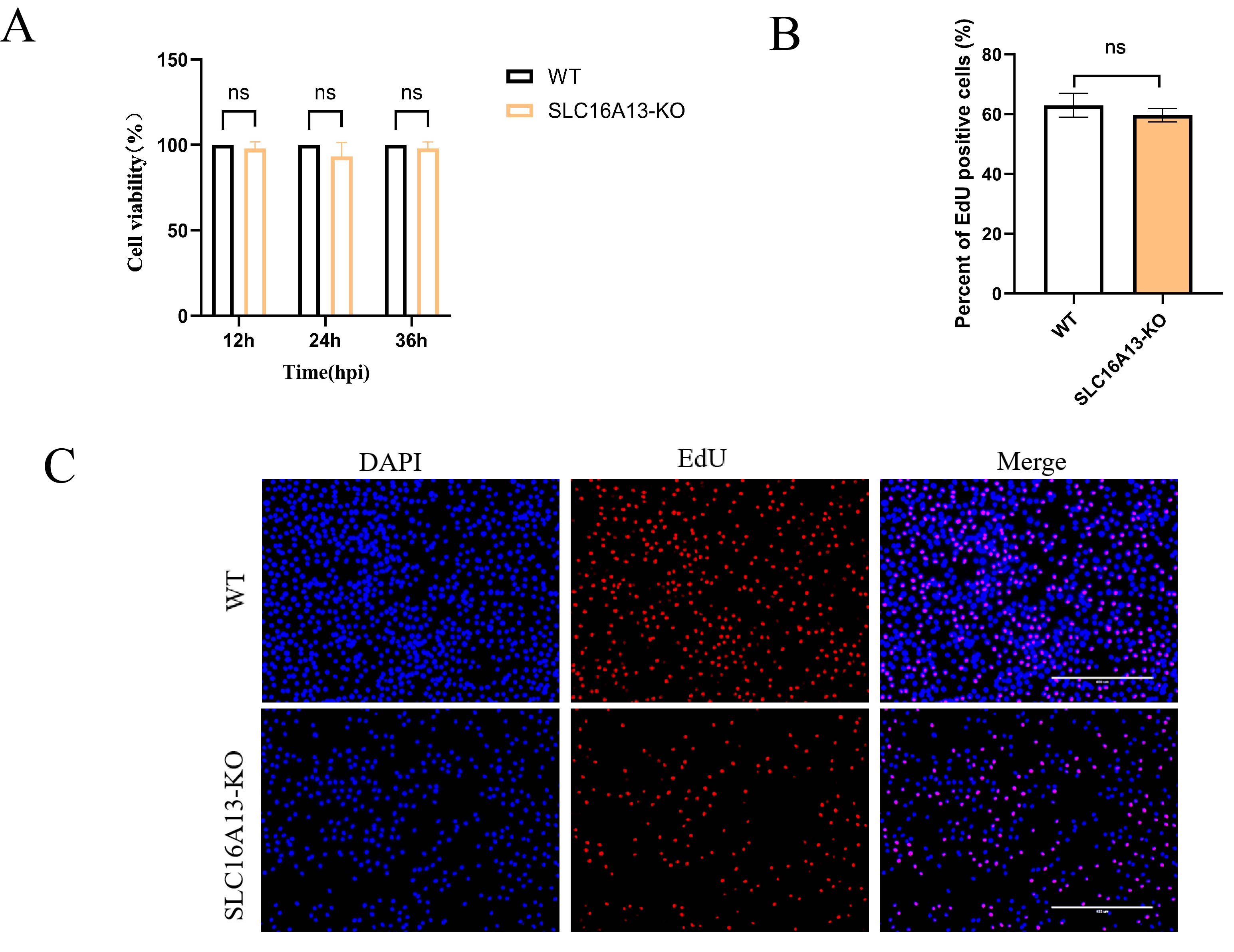


Supplementary Figure 9. Validation of Cell Proliferation Rate and Viability between SLC16A13 KO Cells and WT MDBK Cells. A. Validation of cell viability in SLC16A13 KO cells and WT MDBK cells using a CCK-8 assay. B. Analysis results of EdU cell proliferation assay data. C. Detection of proliferation in SLC16A13 KO cells using an EdU cell proliferation assay. The experimental results were subjected to three technical replications and the representative one of them was presented. Student’s *t*-test for a single comparison and ANOVA for more than one comparison were used. ns, non-significant.
