# Supplementary figures and images for: Genome-scale CRISPR screen identifies host factors associated with bovine parainfluenza virus 3 infection
Source: Virulence. 2025 Dec 2;16(1):2589554. doi: 10.1080/21505594.2025.2589554 (PMC12674325; doi:10.1080/21505594.2025.2589554)

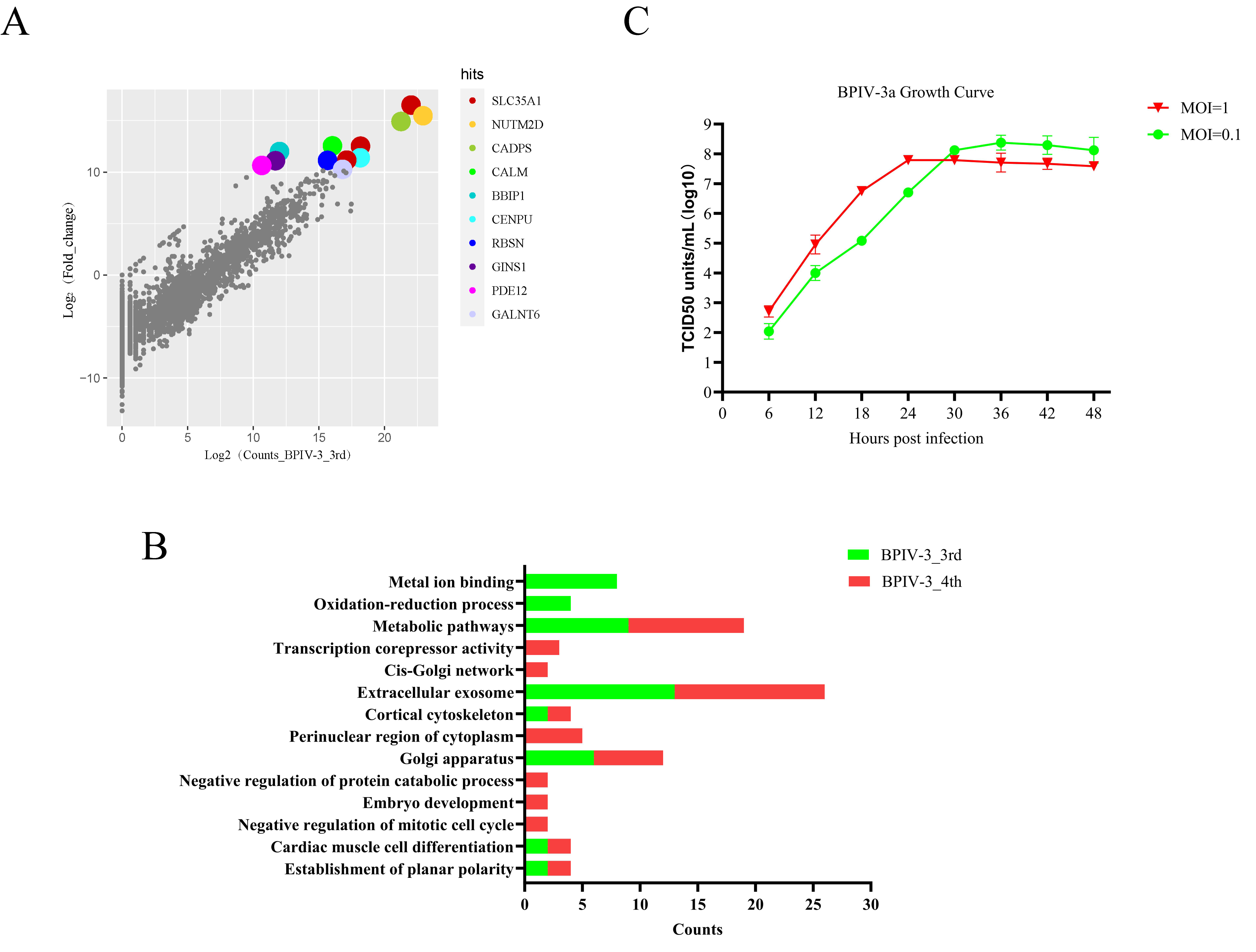

Supplement: Supplementary Figure 3.tif [file KVIR_A_2589554_SM7952.tif]

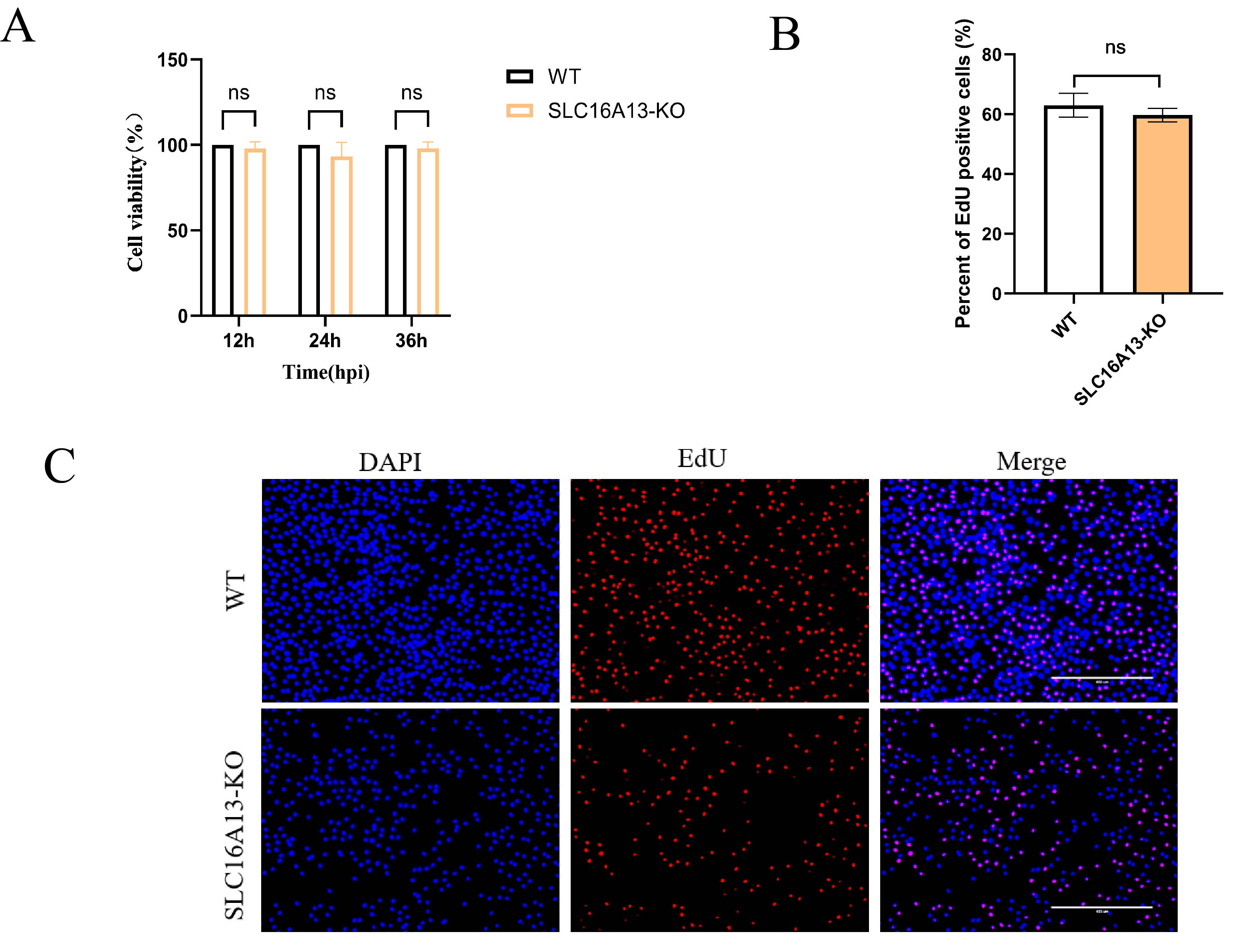

Supplement: Supplementary Figure 9.tif [file KVIR_A_2589554_SM7950.tif]

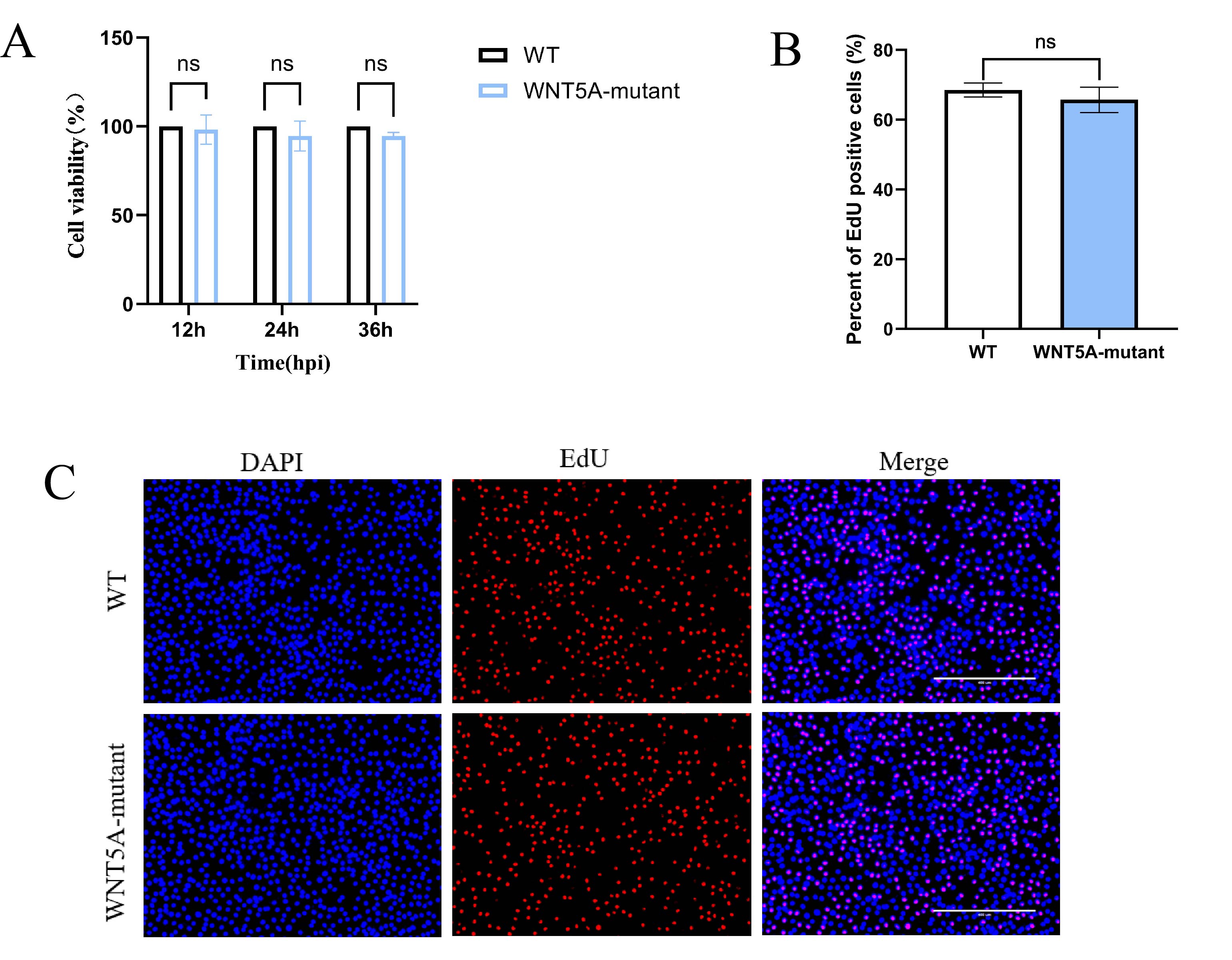

Supplement: Supplementary Figure 6.tif [file KVIR_A_2589554_SM7949.tif]

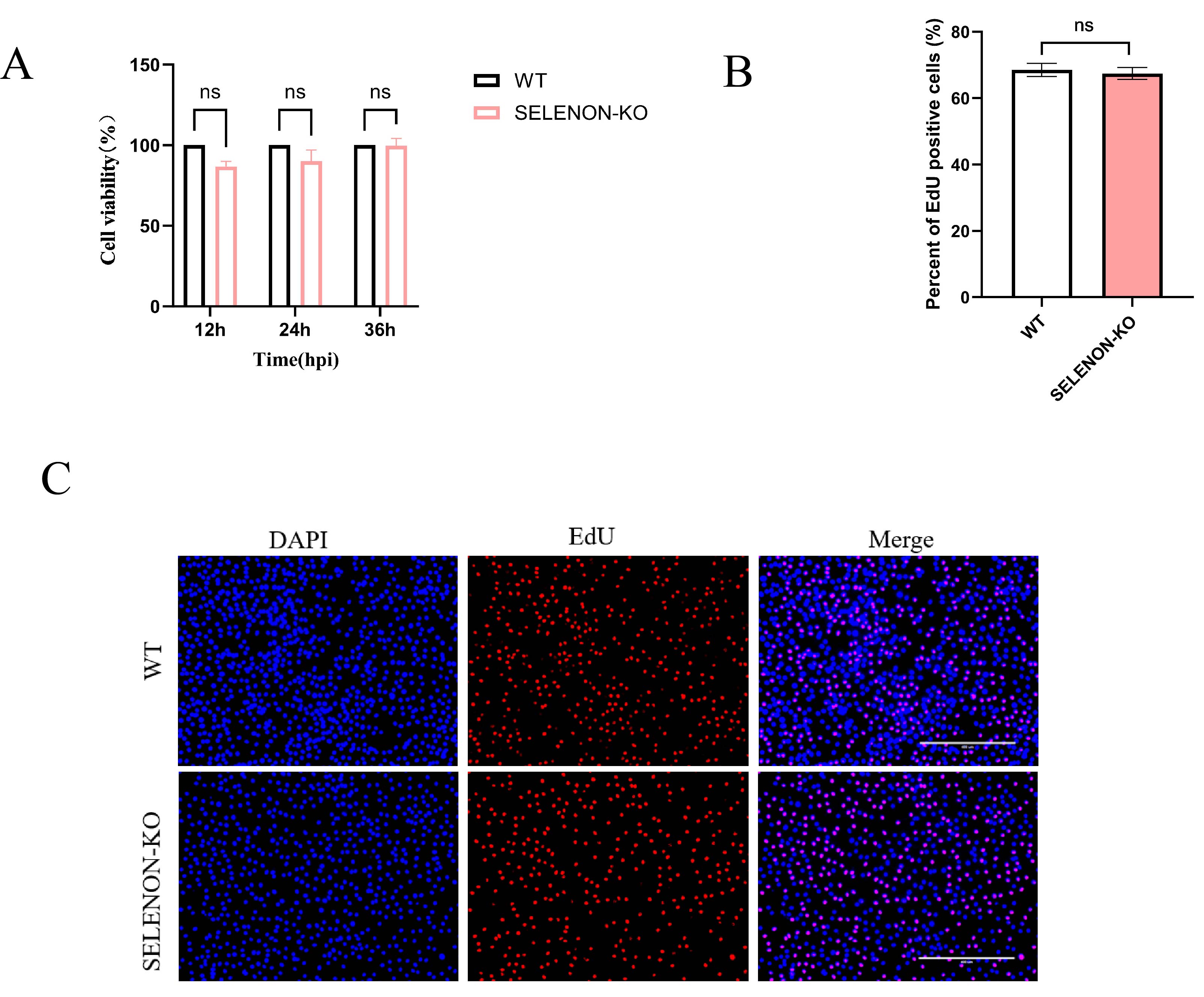

Supplement: Supplementary Figure 8.tif [file KVIR_A_2589554_SM7948.tif]

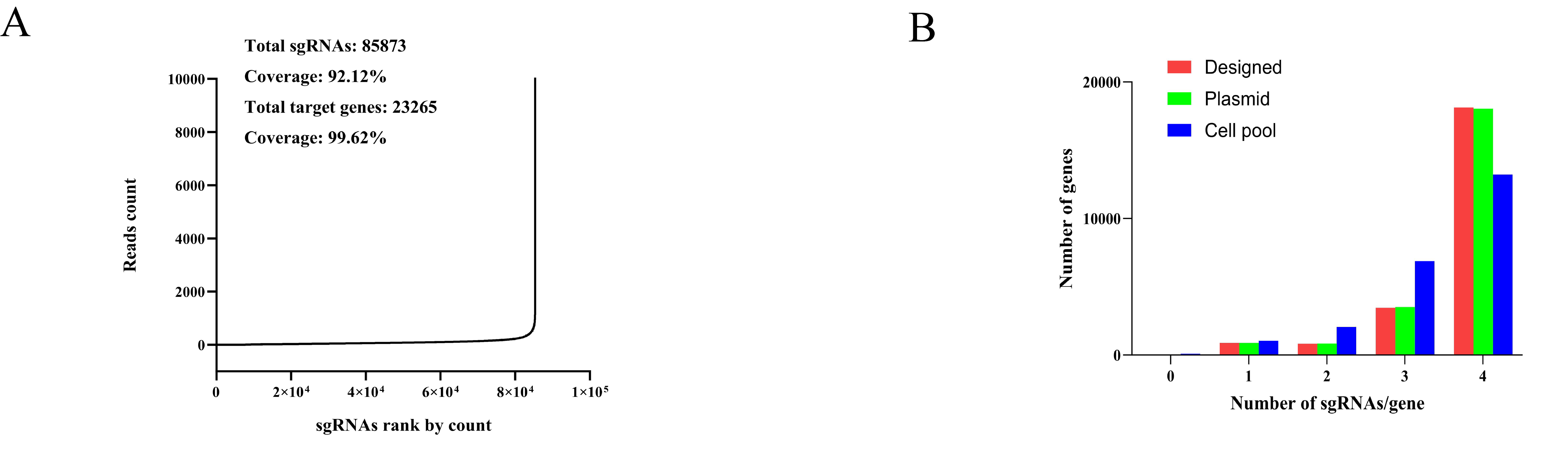

Supplement: Supplementary figure1.tif [file KVIR_A_2589554_SM7947.tif]

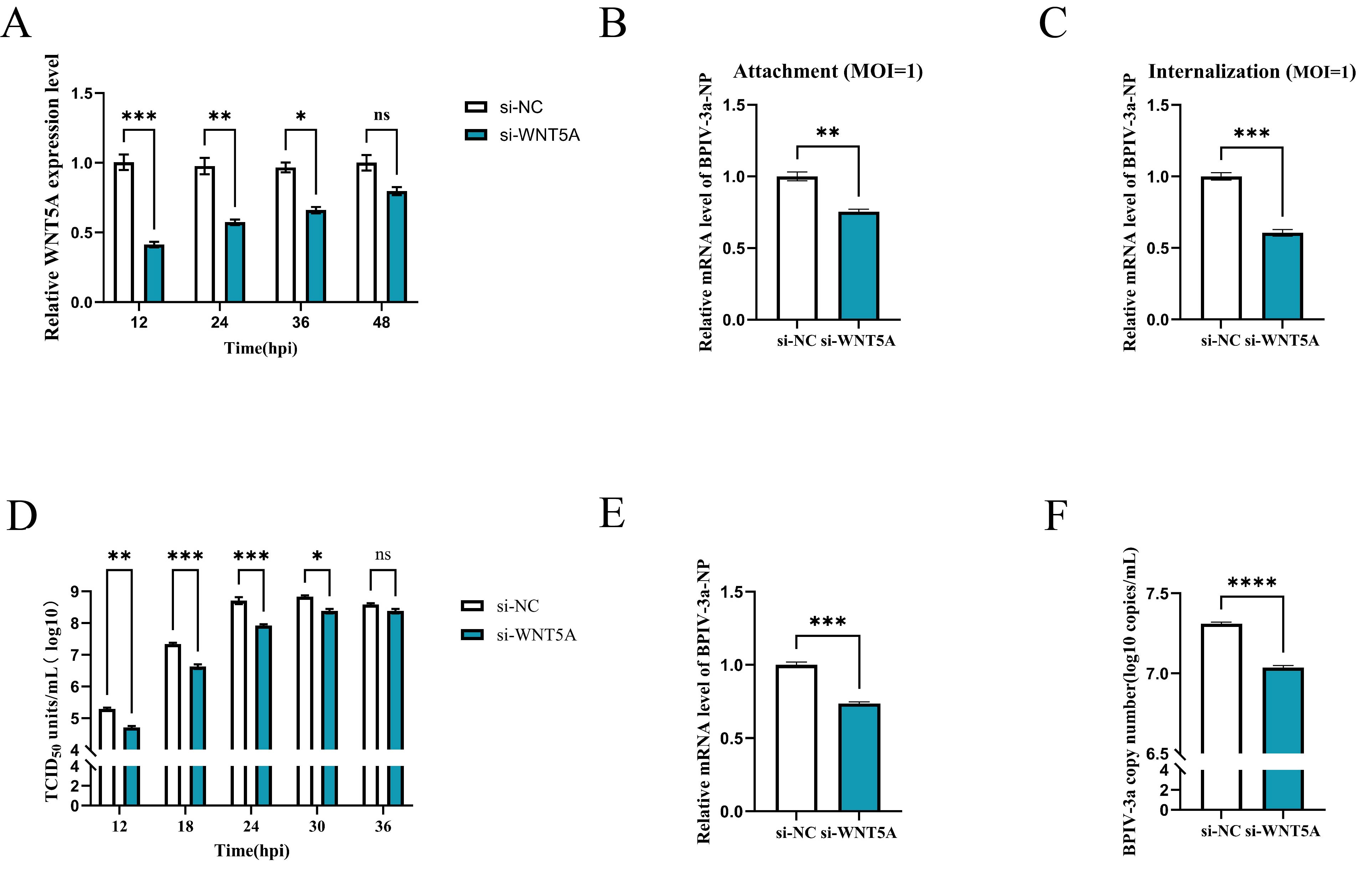

Supplement: supplemental figure 7.tif [file KVIR_A_2589554_SM7946.tif]

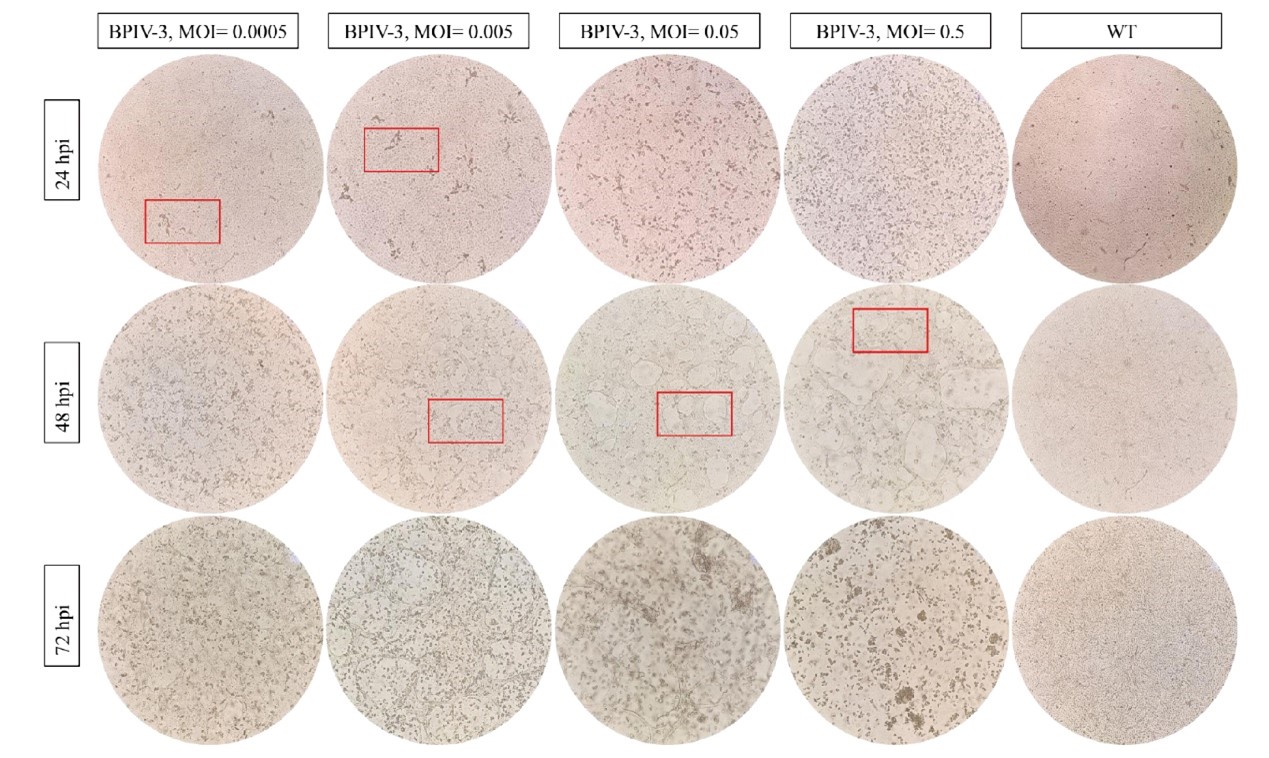

Supplement: Supplementary Figure 2.jpeg [file KVIR_A_2589554_SM7944.jpeg]

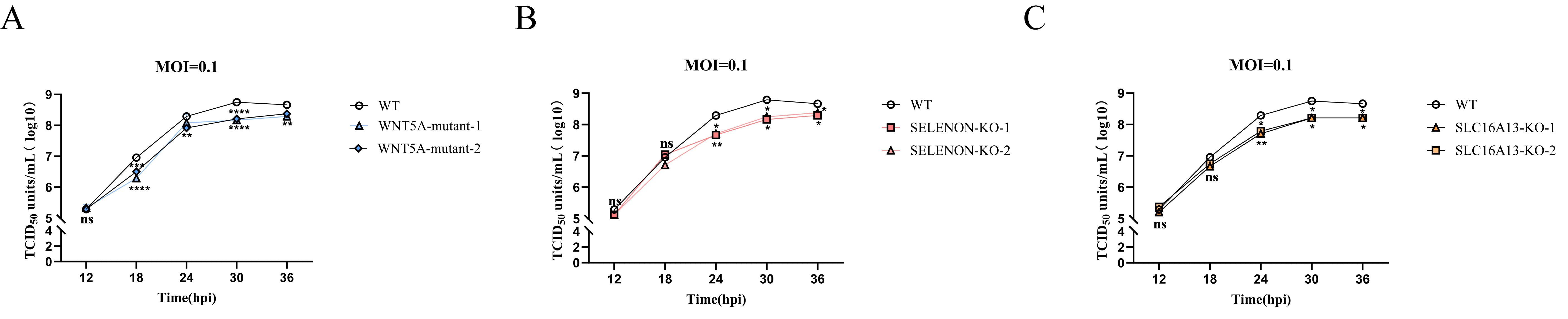

Supplement: supplemental figure 5.tif [file KVIR_A_2589554_SM7943.tif]
